# Coexpression of MmpS5 and MmpL5 Contributes to Both Efflux Transporter MmpL5 Trimerization and Drug Resistance in Mycobacterium tuberculosis

**DOI:** 10.1128/mSphere.00518-20

**Published:** 2021-01-06

**Authors:** Kentaro Yamamoto, Noboru Nakata, Tetsu Mukai, Ikuro Kawagishi, Manabu Ato

**Affiliations:** a Department of Mycobacteriology, Leprosy Research Center, National Institute of Infectious Diseases, Higashimurayama, Tokyo, Japan; b Antimicrobial Research Center, National Institute of Infectious Diseases, Higashimurayama, Tokyo, Japan; c Department of Frontier Bioscience, Hosei University, Koganei, Tokyo, Japan; Washington University School of Medicine in St. Louis

**Keywords:** *Mycobacterium tuberculosis*, bacteriology, bioimaging, multidrug resistance, single-molecule imaging, transporters

## Abstract

The increasing occurrence of multidrug-resistant Mycobacterium tuberculosis (*Mtb*) is a serious threat to global public health. Among the many mechanisms of drug resistance, only resistance-nodulation-division (RND)-type multidrug efflux systems can simultaneously render bacteria tolerant to numerous toxic compounds, including antibiotics. The elevated expression of RND-type xenobiotic efflux transporter complexes, which consist of an inner membrane transporter, membrane fusion protein, and outer membrane channel, plays a major role in multidrug resistance. Among the 14 mycobacterial membrane protein large (MmpL) proteins identified as inner membrane transporters of *Mtb*, MmpL5 is known to participate in the acquisition of resistance to bedaquiline and clofazimine. MmpL5 exports these drugs by forming a complex with the membrane fusion protein mycobacterial membrane protein small 5 (MmpS5). However, the role of MmpS5 in the efflux of antituberculous drugs by MmpL5 remains unclear. In this study, we focused on the *in vivo* dynamics of MmpL5 using green fluorescent protein (GFP). Single-molecule observations of MmpL5 showed substantial lateral displacements of MmpL5-GFP without the expression of MmpS5. Nondiffusing MmpL5-GFP foci typically showed three-step photobleaching, suggesting that MmpL5 formed a homotrimeric functional complex on the inner membrane in the presence of MmpS5. These results suggest that the expression of MmpS5 facilitates the assembly of monomeric MmpL5 into a homotrimer that is anchored to the inner membrane to transport various antimycobacterial drugs.

**IMPORTANCE** It has been reported that mycobacterial membrane protein large 5 (MmpL5), a resistance-nodulation-division (RND)-type inner membrane transporter in Mycobacterium tuberculosis (*Mtb*), is involved in the transport of antimycobacterial drugs. However, the functional roles of the membrane fusion protein mycobacterial membrane protein small 5 (MmpS5), organized as an operon with MmpL5, are unclear. Via the single-molecule imaging of MmpL5, we uncovered the maintenance of the functional trimeric complex structure of MmpL5 in the presence of MmpS5. These findings demonstrate that the assembly mechanisms of mycobacterial RND efflux systems are the dynamically regulated process through interactions among the components. This represents the first report of the single-molecule observation of *Mtb* efflux transporters, which may enhance our understanding of innate antibiotic resistance.

## Abstract (precis)

It has been reported that mycobacterial membrane protein large 5 (MmpL5), a resistance-nodulation-division (RND)-type inner membrane transporter in Mycobacterium tuberculosis (*Mtb*), is involved in the transport of antimycobacterial drugs. However, the functional roles of the membrane fusion protein mycobacterial membrane protein small 5 (MmpS5), organized as an operon with MmpL5, are unclear.

## INTRODUCTION

Tuberculosis (TB), which is caused by Mycobacterium tuberculosis (*Mtb*), is among the deadliest infectious diseases in the world. WHO estimated that TB causes more than 1 million deaths each year (1). Furthermore, the increasing occurrence of multidrug-resistant *Mtb* is a serious health threat. Mycobacteria display passive resistance to harmful compounds, including various antibiotics via their heavy and impermeable cell wall termed the mycomembrane, which has similar functions as the outer membrane of Gram-negative bacteria (2, 3). For convenience, the mycomembrane and the space between it and the inner membrane will be henceforth referred to as the “outer membrane” and the “periplasmic space,” respectively. Most bacteria can acquire antibiotic resistance through active xenobiotic efflux transporters (4). These efflux systems have been classified into five major families based on the similarities of the components, structures, and mechanisms, including energy sources (5). In many Gram-negative bacteria, multidrug resistance is related to the elevated expression of resistance-nodulation-division (RND)-type xenobiotic efflux transporter complexes, which comprise an inner membrane transporter, a membrane fusion protein, and an outer membrane protein (6, 7). As an example of such complexes, the AcrB-AcrA-TolC complex in Escherichia coli has been extensively studied (8). The inner membrane transporter AcrB can capture a wide variety of substrates, including antibiotics, detergents, and other toxic compounds in the periplasmic space and transport them from the cell via the outer membrane protein TolC and membrane fusion protein AcrA using proton motive force (9, 10).

In *Mtb*, 14 genes encoding putative RND-type inner membrane transporters termed mycobacterial membrane protein large (MmpL) genes are involved in metabolic functions, such as siderophore secretions or lipid synthesis, and the transport of various bioactive or harmful substances as well as virulence (11–15). These transporters are predicted to have 12-transmembrane (TM) domains, and their C-terminal residues are located in the cytoplasm. For five of these RND-type transporters, MmpL function requires its cognate mycobacterial membrane protein small (MmpS) protein, which exhibits similarity to RND-type membrane fusion proteins. The genes encoding cognate pairs of MmpLs and MmpSs are organized into operons (16). Thus, it is reasonable to assume that these MmpL-MmpS pairs, like AcrB and AcrA of E. coli, constitute RND-type efflux systems. However, the third component is missing, as no outer membrane protein that can serve as a channel in a manner similar to that of TolC in E. coli has been identified thus far (17). A putative efflux system comprising MmpS5 and MmpL5 contributes to resistance to antimycobacterial drugs, including bedaquiline (BDQ) and clofazimine (CFZ) (18), and to siderophore export (19). Both genes are controlled by a MarR-like transcriptional regulator, which is encoded by the gene Rv0678 located upstream of *mmpS5* (20). A mutation in Rv0678, which increases the expression of both *mmpS5* and *mmpL5*, leads to increased resistance to antimycobacterial drugs (15, 21, 22). Crystallographic analysis of MmpL3 of Mycolicibacterium smegmatis, which is implicated in trehalose monomycolate transport, helped clarify the function of MmpL, including a route through which substrates would follow (23). In the AcrA-AcrB-TolC efflux system of E. coli, it is believed that AcrA stabilizes the interaction between AcrB and TolC (24). The roles of MmpS have been only partially established in mycobacteria. For example, it has been reported that MmpS4 of M. smegmatis acts as a scaffold for the biosynthesis and transport of glycopeptidolipids (25). It is apparent that MmpS of *Mtb* is also a significant component of the RND-type efflux system (26). However, its actual role in drug resistance has not been established.

On the basis of analyses of RND-type efflux pumps in E. coli, we hypothesized that MmpS5 facilitates the assembly of MmpL5 into an efflux pump complex with another as yet unidentified component, i.e., an outer membrane channel. We visualized the MmpL5 dynamics of *Mtb* by tagging MmpL5 with green fluorescent protein (GFP) in Mycobacterium bovis BCG (*mmpL5*, *mmpS5*, and the transcriptional regulator gene are nearly identical between *Mtb* and M. bovis BCG, with identities of 99.8, 100, and 100% at the amino acid level, respectively; see Fig. S1 in the supplemental material). Consistent with our hypothesis, the study results suggested that the expression of MmpS5 facilitates the trimerization of MmpL5 and anchors the trimeric MmpL5 to the peptidoglycan layer or an unknown protein in the outer membrane.

10.1128/mSphere.00518-20.1 FIG S1 Characterization of RND component genes and their similarity at the amino acid level. The loci of RND component genes on the M. tuberculosis and M. bovis BCG Pasteur chromosomes. Download FIG S1, TIF file, 0.8 MB. Copyright © 2021 Yamamoto et al. 2021 Yamamoto et al. This content is distributed under the terms of the Creative Commons Attribution 4.0 International license.

## RESULTS

### The inner membrane transporter MmpL5 requires its cognate membrane fusion protein MmpS5 to confer resistance to multiple antimycobacterial drugs.

To examine the effect of MmpS5 on MmpL5 activity in *Mtb*, we constructed an M. bovis BCG strain (NNB001) in which their homolog genes were deleted (ΔBCG_0727-*mmpSL5*). We also constructed integrative plasmids encoding GFP-fused MmpL5 and nonfused full-length MmpL5 with or without MmpS5. NNB001 cells were transformed with these plasmids, and the resulting cells expressed MmpL5-GFP under the control of the *hsp60* promoter in the vector. The MICs of BDQ, CFZ, and clarithromycin (CLR) in each strain were determined in the presence or absence of MmpS5 (Table 1). The strain expressing MmpL5 with MmpS5 exhibited substantially higher MICs for BDQ (32-fold) and CFZ (4-fold), but not for CLR, than the strain carrying the empty vector. In contrast, MmpL5 had no effect on the MICs of BDQ and CFZ in the absence of MmpS5. Furthermore, the MmpL5-GFP protein retained almost full activity with MmpS5 as judged by the MICs. Consistent with this, immunoblotting using anti-GFP antibody detected no visible degradation product (see Fig. S2 in the supplemental material).

**TABLE 1:** Antibiotic susceptibility analysis^*a*^

Drug	MIC (μg ml^−1^)					
	Δ*mmpSL5*					WT, vector
	L5	L5-GFP	S5-L5	S5-L5-GFP	Vector	
BDQ	0.125	0.125	**4**	**4**	0.125	**2**
CFZ	0.25	0.25	**1**	**1**	0.25	**1**
CLR	0.16	0.16	0.16	0.16	0.16	0.16

a The integrated vector-encoded wild-type MmpL5 and GFP-fused MmpL5 with or without MmpS5 were expressed in strain NNB001 (Δ*mmpS5-L5-*BCG_0727::*Hyg*). Values shown in boldface type represent resistance indicative of significant efflux activity. Abbreviations: BDQ, bedaquiline; CFZ, clofazimine; CLR, clarithromycin.

10.1128/mSphere.00518-20.2 FIG S2 Detection of MmpL5-GFP using immunoblotting. MmpL5-GFP (*m*/kDa = 133.5) was expressed in NNB001 strain carrying the MmpS5-MmpL5-GFP (SL5-GFP) or MmpL5-GFP expression plasmid (L5-GFP). The BCG wild-type strain (WT) and NNB001 strain carrying the free-GFP expression plasmid (GFP, *m*/kDa = 28) were used as controls. Whole-cell lysates were subjected to Western blotting using a monoclonal antibody raised against GFP and an anti-mouse IgG antibody labeled with horseradish peroxidase. Download FIG S2, JPG file, 0.07 MB. Copyright © 2021 Yamamoto et al. 2021 Yamamoto et al. This content is distributed under the terms of the Creative Commons Attribution 4.0 International license.

### MmpS5 appears to restrict the lateral diffusion of MmpL5 in the inner membrane.

**FIG 1:**
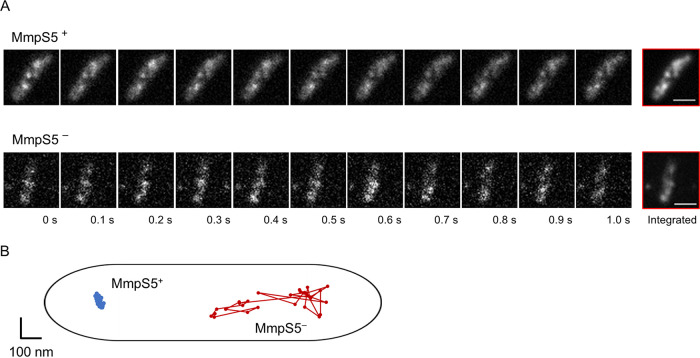
Dynamics of GFP-labeled MmpL5 in the inner membrane. (A) Real-time observation of MmpL5-GFP foci using TIRF microscopy. The BCG_0727-*mmpS5*-*mmpL5* deleted strains were complemented by pKRB32 or pKRB29. These cell images present the dynamics of the fluorescent foci of MmpL5-GFP in the presence (top) or absence (bottom) of MmpS5. Data were acquired at 33 ms per frame. Bars, 1 µm. (B) The *x*-*y* trajectories of MmpL5-GFP in the presence (blue line) or absence (red line) of MmpS5.

**FIG 2:**
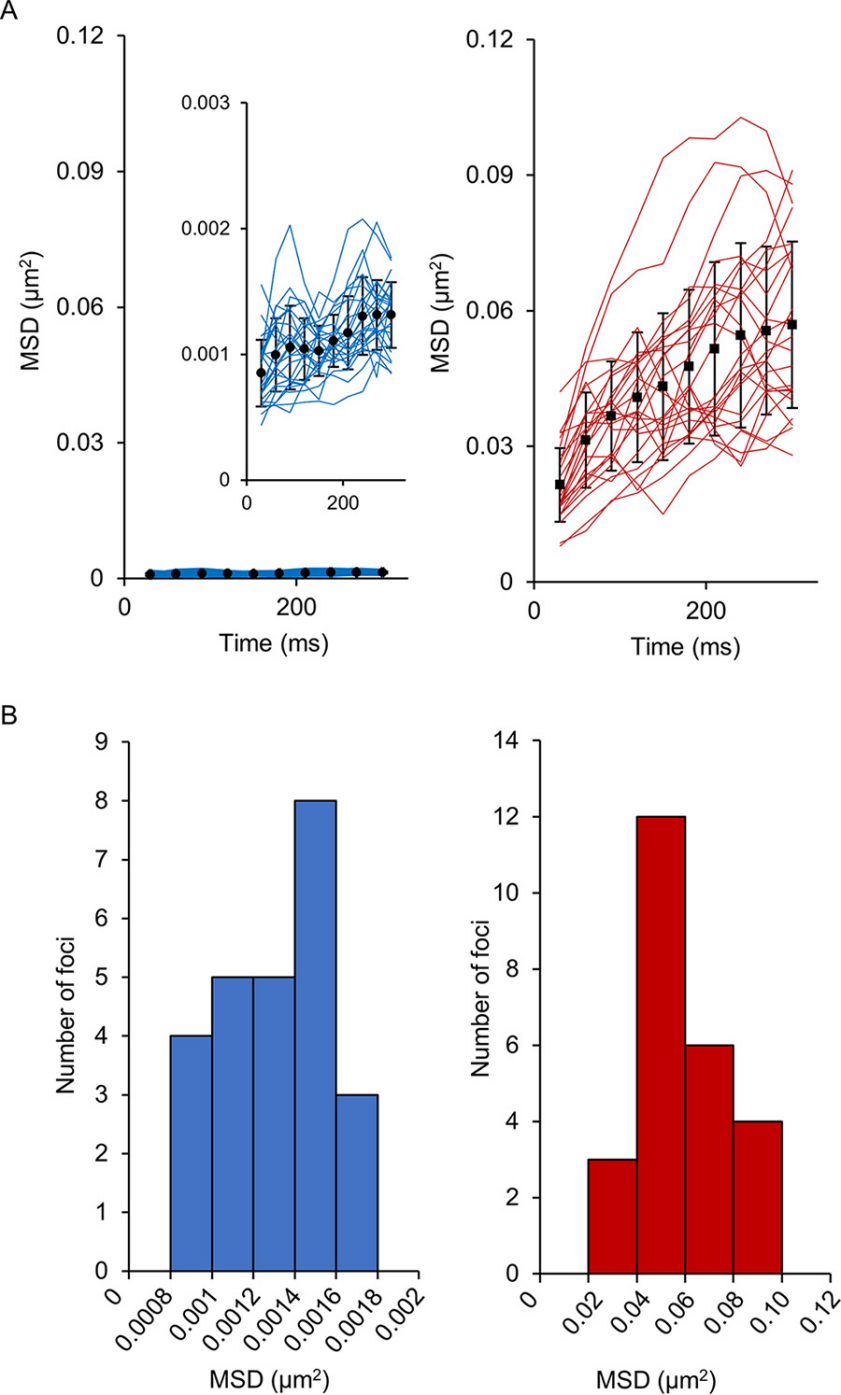
MSD of GFP-labeled MmpL5 using TIRF microscopy. Calculation of the MSD of MmpL5-GFP in the presence (blue line and bar) or absence (red line and bar) of MmpS5 (*n *= 25). (A) MSD-Δ*t* plots of their foci. The closed symbols with error bars show the standard deviations of MSDs with all fixed (circles) or mobile (squares) foci. (Inset) The small graph presents the MSD-Δ*t* plots of fixed MmpL5-GFP foci using a different *y* axis scale. (B) Distribution of the MSD of MmpL5-GFP foci at 330 ms.

To examine the effect of MmpS5 on the behavior of MmpL5-GFP foci in the inner membrane of mycobacteria, single-molecule observation was conducted using total internal reflection fluorescence (TIRF) microscopy (Fig. 1 and 2). MmpL5-GFP foci underwent extremely limited lateral displacement in the presence of MmpS5, whereas they diffused laterally throughout the inner membrane in the absence of MmpS5 (Fig. 1A; see also Movies S1 and S2 in the supplemental material). The *x*-*y* trajectories of MmpL5-GFP foci in these backgrounds indicate that MmpL5 diffuses randomly along the membrane (Fig. 1B). The range of movement of MmpL5-GFP fluorescent foci was expressed as the two-dimensional mean square displacement (MSD) of individual foci. In the presence of MmpS5, MmpL5-GFP movement was restricted, and the calculated MSDs of the foci at 330 ms were distributed in the range of 0.08 × 10^−2^ to 0.18 × 10^−2^ µm^2^ with a mean value of 0.13 × 10^−2^ ± 0.03 × 10^−2^ µm^2^ (Fig. 2A and B, blue lines and bars). In the absence of MmpS5, most MmpL5-GFP foci moved incessantly. Their MSDs ranged widely (2.80 × 10^−2^ to 9.10 × 10^−2^ µm^2^) with a mean value of 5.69 × 10^−2^ ± 1.84 × 10^−2^ µm^2^ (Fig. 2A and B, red lines and bars). The diffusion coefficients (*D*) in the presence and absence of MmpS5 as calculated from these MSD-Δ*t* plots were 5.8 × 10^−4^ ± 2.2 × 10^−4^ and 3.4 × 10^−2^ ± 1.7 × 10^−2^ µm^2^ s^−1^, respectively. We then observed MmpL5-GFP foci in the presence of the BCG_0727 gene (strain YKN80) encoding the hypothetical transcriptional regulator. The data illustrated that the diffusion of MmpL5-GFP is also restricted under the expression of MmpS5 regardless of the presence of the putative transcriptional regulator gene (Movie S3). On the basis of these data, MmpL5-GFP foci were defined as “fixed (immobile)” in the presence of MmpS5 and “mobile” in the absence of MmpS5.

10.1128/mSphere.00518-20.5 MOVIE S1 Dynamics of MmpL5-GFP foci in the presence of MmpS5. This movie presents the dynamics of MmpL5-GFP in the presence of MmpS5 on the inner membrane of NNB001 cells using TIRFM. Data were recorded at 30 frames per second. Bar, 1 µm. Download Movie S1, AVI file, 0.6 MB. Copyright © 2021 Yamamoto et al. 2021 Yamamoto et al. This content is distributed under the terms of the Creative Commons Attribution 4.0 International license.

10.1128/mSphere.00518-20.6 MOVIE S2 Dynamics of MmpL5-GFP foci in the absence of MmpS5. This movie presents the dynamics of MmpL5-GFP in the absence of MmpS5 on the inner membrane of NNB001 cells using TIRFM. Data were recorded at 30 frames per second. Bar, 1 µm. Download Movie S2, AVI file, 0.5 MB. Copyright © 2021 Yamamoto et al. 2021 Yamamoto et al. This content is distributed under the terms of the Creative Commons Attribution 4.0 International license.

10.1128/mSphere.00518-20.7 MOVIE S3 Dynamics of MmpL5-GFP foci in the presence of MmpS5 and the BCG_0727 gene. This movie shows the dynamics of MmpL5-GFP in the presence of MmpS5 and the BCG_0727 gene, which is a MarR-like transcriptional regulator that downregulates the expression of *mmpS5-L5*, on the inner membrane of YKN80 cells using TIRFM. Data were recorded at 30 frames per second. Bar, 1 µm. Download Movie S3, AVI file, 0.8 MB. Copyright © 2021 Yamamoto et al. 2021 Yamamoto et al. This content is distributed under the terms of the Creative Commons Attribution 4.0 International license.

### Fixed MmpL5 forms a homotrimer in the presence of MmpS5.

**FIG 3:**
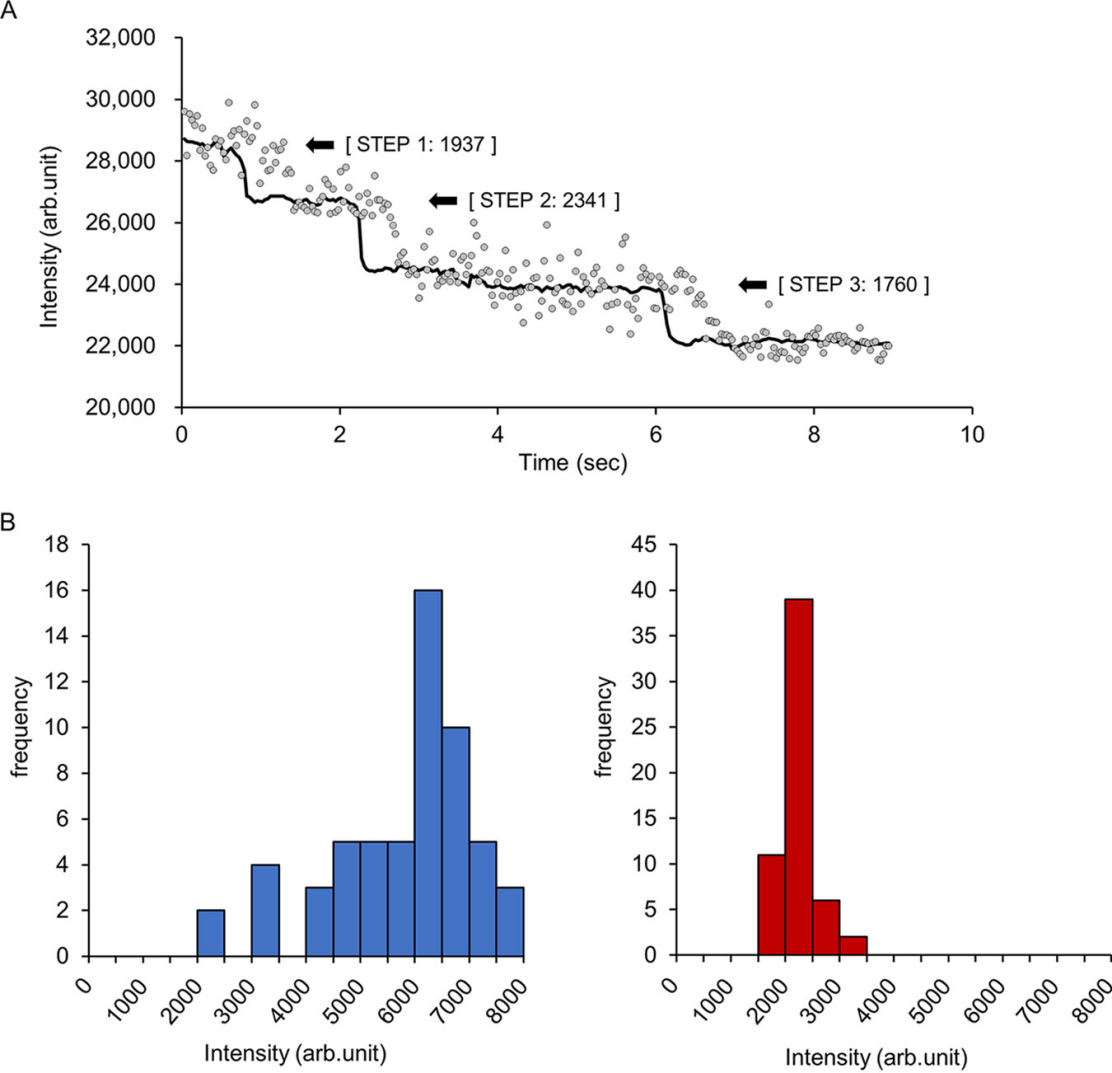
Estimation of the number of GFP-labeled MmpL5 molecules in a single focus. (A) Stepwise photobleaching of single molecules in MmpL5-GFP foci in the presence of MmpS5. Gray dots show the intensity-Δ*t* plots of MmpL5-GFP. Each step was identified using the edge detection filtered intensity (black line). The black arrows indicate the positions of predicted bleaching steps, and numbers indicate the intensity of the detected steps. Intensity is shown in arbitrary units. (B) Distribution plots of the fluorescent intensity of MmpL5-GFP at the first frame in the presence (blue bar) or absence (red bar) of MmpS5 (*n *= 58).

To ascertain whether fixed MmpL5-GFP foci consist of single or oligomeric molecules, we performed time course analyses of the fluorescence emission from fixed single MmpL5-GFP foci. The filtered intensity-Δ*t* plots calculated using edge detection methods indicated a three-step photobleaching plot (Fig. 3A). The step size of a single GFP molecule was equivalent to 2,012 ± 243 (arbitrary units) under these experimental conditions. In the presence of MmpS5, the distribution of intensity value at the first frame peaked at 6,000 to 6,500, which was approximately threefold higher than the intensity of single GFP molecules estimated using photobleaching (Fig. 3B). In the absence of MmpS5, that value peaked at 2,000 with a narrow range. These results indicate that MmpL5-GFP forms a homotrimer in the presence of MmpS5.

### Cell wall morphology is not affected by deletion of *mmpS5* or *mmpL5*.

**FIG 4:**
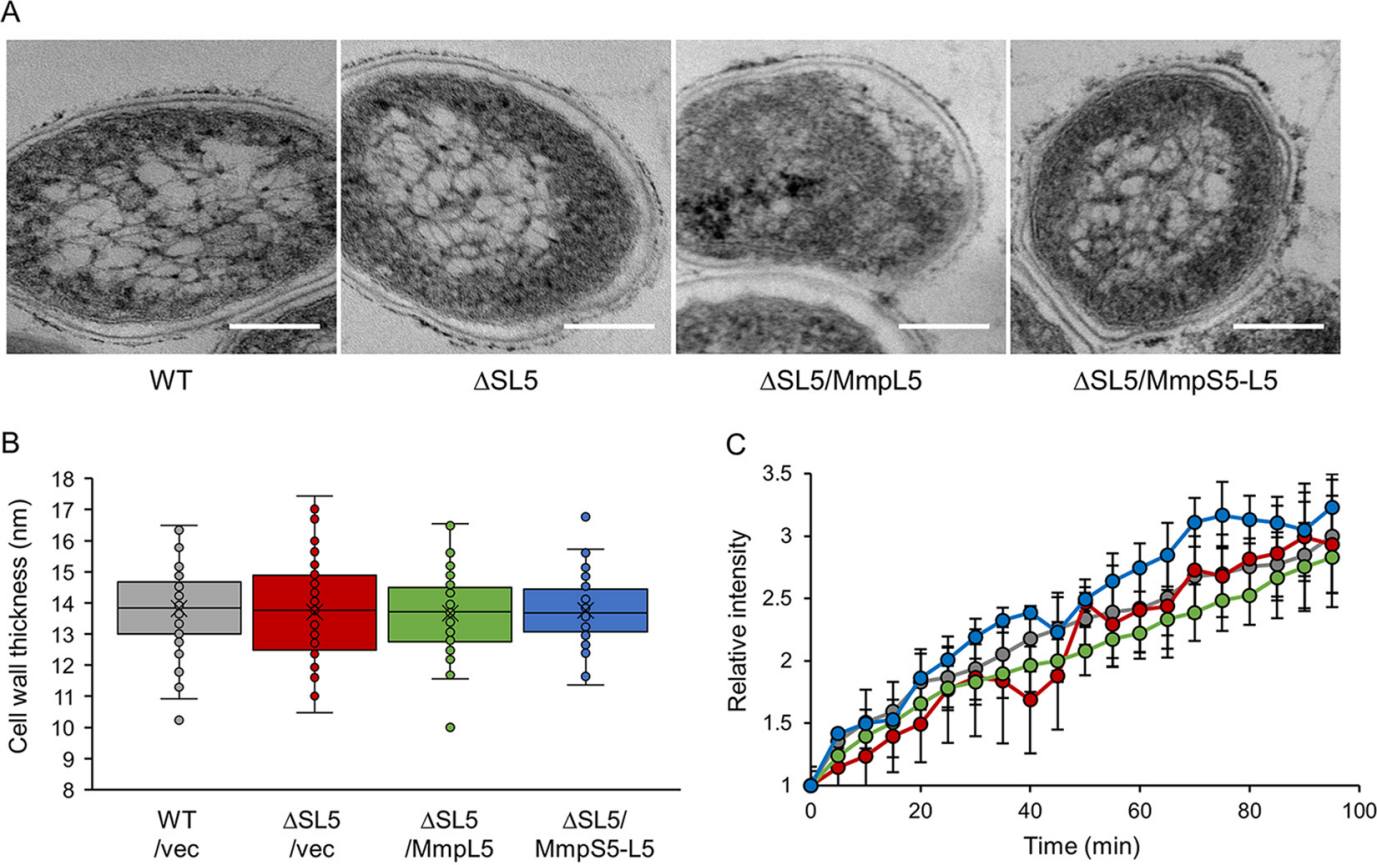
Cell wall morphology under each strain background. Analysis of Mycobacterium bovis BCG cell shape, cell wall thickness, and permeability. WT, wild-type; ΔSL, *mmpS5-mmpL5*-deleted strain. (A) Transmission electron micrographs of BCG strains. Bars, 100 nm, Magnification, ×30,000. (B) Dot plots showing cell wall thickness (*n *= 52) of various BCG strains transfected with empty vectors (vec), pKRB29 (/MmpL5), and pKRB32 (/MmpS5-L5). (C) Time course of the relative intensity of intracellularly accumulated ethidium bromide in the presence of verapamil (*n *= 3). Colors of dots are consistent with each strain background in panel B.

We then examined whether the deletion of *mmpS5* and *mmpL5* influenced cell wall morphology using transmission electron microscopy (TEM). During the logarithmic growth phase, the cell shapes of wild-type and *mmpS5-* and *mmpL5*-deleted strains exhibited no significant differences (Fig. 4A). The mean cell wall thickness of wild-type BCG was 13.82 ± 1.29 nm, versus 13.71 ± 1.59 nm in the absence of MmpS5 (Fig. 4B). We also calculated cell wall thickness of strains expressing MmpL5-GFP or MmpS5-MmpL5-GFP in the absence of MmpS5 (Fig. 4B). The mean values were 13.67 ± 1.28 and 13.82 ± 1.07 nm, respectively (*P* > 0.78). Furthermore, to investigate whether the presence of MmpL5 and/or MmpS5 affects the permeability of the cell wall, we evaluated the accumulation rate of the fluorescent compound ethidium bromide (EtBr) following treatment with the efflux pump inhibitor verapamil by measuring the fluorescence intensity (Fig. 4C). We found no difference in the ratio of fluorescence intensity in each background. These results suggest that the *mmpS5* or *mmpS5-mmpL5* deletion does not affect cell wall morphology.

## DISCUSSION

Some RND-type efflux systems appear to participate in antimicrobial resistance in mycobacteria by discharging harmful environmental compounds (27). In this work, we focused on MmpS5 and MmpL5, which are supposed to be an inner membrane transporter and its cognate membrane fusion protein, to export CFZ and BDQ (18, 28, 29). Because the membrane fusion protein-like protein MmpS5 granted MmpL5 the ability to export substances (26), it has been postulated that MmpS5 may be an essential part of the efflux pump involved in drug transport. However, this had not been demonstrated previously. Using an *mmpS5 mmpL5* knockout strain with complementation of the *mmpS5* and/or *mmpL5* genes, we demonstrated that coexpression of MmpS5 and MmpL5 is essential for the acquisition of BDQ and CFZ resistance.

Fluorescent imaging with GFP showed that the localization of MmpL5 appeared to be arranged in a punctiform pattern in the presence of MmpS5. In E. coli, the membrane fusion protein AcrA is anchored to the inner membrane via a lipid moiety (30). It is possible that MmpS5, a homolog of AcrA, is associated with specialized microdomains in the inner membrane, such as membrane rafts or those demarcated by specific cytoskeleton-like proteins. Alternatively, a putative outer membrane channel could be confined to some kind of microdomains in the outer membrane.

The dynamics of MmpL5-GFP with or without MmpS5 revealed a substantial difference between these backgrounds in the physical limitation of MmpL5 mobility in the membrane. Deletion of MmpS5 granted MmpL5 mobility in the inner membrane. A previous study indicated that the RND-type transporter AcrB of E. coli, in the absence of the outer membrane channel TolC, exhibited lateral displacement without being trapped in the membrane (31). *D* of mobile MmpL5 was consistent with that reported previously for mobile GFP-fused AcrB (*D*_mean_ = 3.5 × 10^−2^ µm^2^ s^−1^). The magnitude of the diffusion of membrane protein depends on the size of the TM domain. Specifically, *D*_mean_ of 4-TM helical proteins was 2.0 × 10^−1^ µm^2^ s^−1^ compared with 2.0 × 10^−2^ µm^2^ s^−1^ for 12-TM helical proteins (32). These data confirmed that both MmpL5 and AcrB have 12-TM domains and similar molecular masses (molecular mass in kilodaltons [*m/*kDa] = 104.8 versus 113.6). Only a few studies have assessed the diffusion of Gram-positive bacteria membrane proteins, mainly including studies in Bacillus subtilis (33, 34). Mika et al. reported that *D* of GFP-fused BcaP, which is an amino acid transporter in Lactococcus lactis, was 1.9 × 10^−2^ µm^2^ s^−1^ (35), consistent with that observed for MmpL5 in the present study. The fact that MmpL5-GFP foci are fixed in the presence of MmpS5 indicates that MmpL5 is anchored to a nondiffusing structure, an unknown hypothetical channel-like outer membrane protein, penetrating both the cell wall and the outer membrane (i.e., the mycomembrane) (9). The peptidoglycan layer can be predicted to have an intricate three-dimensional mesh-like framework including arabinogalactan (36). This rigid layer restricts the lateral diffusion of MmpL5 binding to the layer or a penetrated outer membrane protein. Thus, the diffusion of MmpL5 must also be restricted via assembly into a complex in the presence of MmpS5, which leads to the acquisition of drug efflux activity. However, it remains to be elucidated whether MmpL5 is stabilized only through its interaction with MmpS5 or with the help of other factors.

In this study, MmpL5-GFP foci were revealed to be fixed by the expression of MmpS5 in the presence of the BCG_0727 gene, which has an extremely high similarity with Rv0678 of *Mtb* (100% identity at the amino acid level), encoding the conserved hypothetical MarR-like transcriptional regulator, which downregulates *mmpS5-L5* expression (28, 29). This finding indicates that this regulator does not affect the dynamics of MmpL5 by regulating the expression of some unknown genes.

We examined the three-step photobleaching of fixed MmpL5-GFP foci in the presence of MmpS5. The distribution plot of focus intensity in the first frame revealed that MmpL5 predominantly forms a homotrimer in the presence of MmpS5, whereas it remains a monomer in the absence of MmpS5. MmpL3 of *Mtb*, a paralog of MmpL5, was suggested to be a monomer according to a blue native polyacrylamide gel electrophoresis (BN-PAGE) analysis with purified MmpL3 from cultured M. smegmatis harboring an MmpL3-expressing plasmid (23). However, this apparent discrepancy was not surprising as the authors discuss the possibility that the membrane fusion protein-like protein MmpS3, which is missing in their experimental setting, may play a role in trimerization of MmpL3. Thus, we propose that MmpS5 contributes to the ability of MmpL5 to form a fixed active homotrimer in the membrane.

These findings will help understand the drug resistance of the MmpS-MmpL transporting systems in *Mtb* as well as in other mycobacteria. Indeed, recent studies have reported that the orthologs of MmpS5 and MmpL5 of Mycobacteroides abscessus are also involved in CFZ and BDQ resistance, which is regulated by transcriptional regulators, such as the MarR-like transcriptional regulator of *Mtb* (37, 38).

Mycobacterial cell walls have complex structures containing a lipid-rich surface of mycolic acid, glycolipids, and other polymers. Some RND-type pumps, including MmpL3 and MmpL11, are involved in metabolism of these cell wall components (39, 40). However, it is unknown whether MmpS5 and MmpL5 transport cell wall components. The effects of the expression of RND-type pumps on cell morphology and physiology have not been studied. The cell wall thickness in each background of the BCG strain was extremely similar and consistent with that reported for the *Mtb* cell wall (mean thickness = 13.93 nm) (41). The cell envelope structure of mycobacteria is substantially different from that of Gram-negative bacteria: unlike the outer membrane of the latter, mycobacteria have a mycomembrane comprising a nonconventional lipid bilayer in which the inner leaflet contains very long-chain fatty acids, named mycolic acids, covalently linked to arabinogalactan, a component of the thick peptidoglycan layer. These unique features might render the channel components considerably diversified from those of Gram-negative bacteria, such as TolC of E. coli, and hence elusive from bioinformatic identification. Moreover, the cell wall permeability of EtBr was not affected by the altered expression of MmpS5-L5. Although the membrane efflux transporter P55 of *Mtb* transports EtBr (42), the calcium channel antagonist verapamil was used to inhibit the EtBr efflux activities of P55 (43, 44). Similar levels of EtBr accumulation in various backgrounds suggest that the differences in the motility of MmpL5 are not attributable to structural changes in the cell wall architecture.

**FIG 5:**
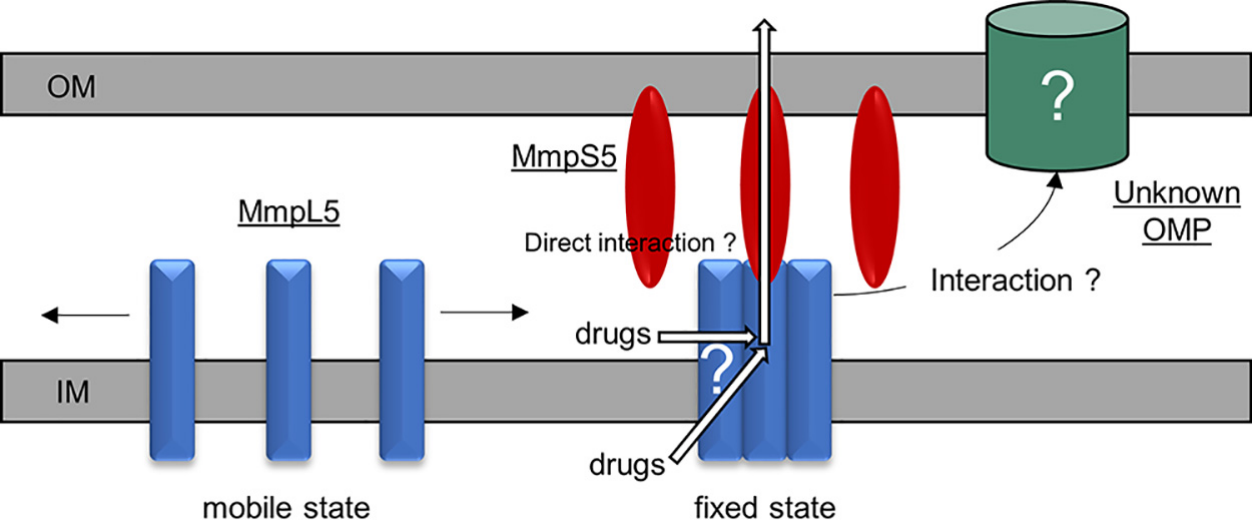
Model of the existence of MmpS5-mediated anchoring of MmpL5 to the outer membrane of Mycobacterium tuberculosis. Monomeric MmpL5 is freely mobile on the inner membrane (IM). MmpS5 facilitates the assembly of MmpL5 into a homotrimer and anchors the trimeric MmpL5 to the outer membrane (OM) or an unknown hypothetical outer membrane protein (OMP). For simplicity, the peptidoglycan layer is abbreviated, and MmpS5 is depicted to bind MmpL5 evenly. Two putative pathways of drug entry into MmpL5 are shown.

In conclusion, we found that the expression of the membrane fusion protein MmpS5 facilitates the homotrimerization of MmpL5 and restricts its diffusion presumably due to the assembly into a ternary complex with a yet unidentified outer membrane channel component to export anti-*Mtb* drugs. The summarized model is depicted in Fig. 5. More detailed studies are required to delineate the interaction between MmpS and MmpL and investigate whether MmpS5 anchors the transporter complex to a specific outer membrane protein or directly to the mycomembrane. It is likely that the behavior of MmpL5 in the presence of MmpS5 can also be observed in all cognate MmpS-MmpL pairs in mycobacteria. Therefore, the homotrimerization of MmpL and the anchoring of the MmpL/MmpS complex to the mycomembrane may be promising targets for new anti-*Mtb* drugs.

## MATERIALS AND METHODS

### Strains, plasmids, primers, and culture condition.

The bacterial strains, plasmids, and primers used in this study are described in Table S1 in the supplemental material. M. bovis BCG Pasteur strains were grown at 37°C under shaking conditions in enriched 7H9 medium (Middlebrook 7H9 medium supplemented with 0.2% glycerol, 0.05% Tween 80, and albumin-dextrose-catalase [ADC] enrichment) or on enriched 7H10 agar (Middlebrook 7H10 supplemented with 0.5% glycerol and oleic acid-albumin-dextrose-catalase [OADC] enrichment). Whenever necessary, 50 µg ml^−1^ kanamycin or 100 µg ml^−1^ hygromycin was added to the medium for clone selection.

10.1128/mSphere.00518-20.3 TABLE S1 Bacterial strains and plasmids used in this study. Download Table S1, DOCX file, 0.03 MB. Copyright © 2021 Yamamoto et al. 2021 Yamamoto et al. This content is distributed under the terms of the Creative Commons Attribution 4.0 International license.

### Plasmids.

The plasmid vector pNN301 is a pMV361-type E. coli-*Mycobacterium* shuttle vector that carries the *hsp60* promoter, integrase gene, and kanamycin resistance gene. In addition, the vector contains the *attP* region for integration into the *attB* site of mycobacterial chromosomal DNA (45, 46).

### Construction of plasmids encoding GFP and/or GFP-fused MmpL5.

The *gfp* coding region was amplified by PCR using pDS1050 containing Gly_3_-GFP to introduce SalI and HpaI sites at their 5′ and 3′ ends, respectively. The resulting fragment was cloned between the SalI and HpaI sites of the pNN301 vector to yield plasmids encoding GFP (pKRB1). For GFP-fused MmpL5 expression, pKRB1 plasmids were used as vectors to clone a PCR-amplified MmpL5 or MmpS5-MmpL5 operon region in-frame in the chromosomal DNA of *Mtb* H37Rv with EcoRI and ClaI sites at its 5′ and 3′ ends, respectively, thereby yielding pKRB29 and pKRB32, respectively. The wild-type MmpL5-expressing plasmids (pKRB30 and pKRB34) were constructed using the same cloning strategy.

### Construction of gene deletion strains.

The allelic exchange strain NNB001 (ΔBCG_0727-*mmpSL5*) was constructed using a temperature-sensitive mycobacteriophage method as described previously (46–48). Briefly, the upstream and downstream flanking DNA sequences in the M. bovis BCG genome sequence (GenBank accession number AM408590) were used to delete the BCG_0725c (*mmpL5*), BCG_0726c (*mmpS5*), and BCG_0727 genes. To disrupt these three genes, DNA segments from 935 bp upstream through 5 bp downstream of the termination codon of *mmpL5* and from 4 bp through 1003 bp downstream of the termination codon of BCG_0727 were used.

The Δ*mmpSL5* strain was constructed using the Che9c recombination system with pJV53 plasmids encoding *gp60* and *gp61*, which are responsible for exonuclease and DNA-binding activities, respectively (49). To replace the operon of *mmpS5* or *mmpL5* in the chromosome of M. bovis BCG carrying the selectable hygromycin resistance gene, the DNA fragments were generated by PCR using the pYUB854 plasmid as the template, which is the hygromycin resistance cassette containing 1,000-nucleotide homology extensions, upstream or downstream of the *mmpSL5* operon. After selection on enriched 7H10 agar containing 100 µg ml^−1^ hygromycin, recombinants were purified twice. Replacement of these genes in each chromosome of M. bovis BCG mutants was confirmed by PCR.

### Antibiotic susceptibility analysis.

MICs were determined using a twofold serial dilution method (50, 51). Precultured cells in the mid-logarithmic phase were diluted at an optical density at 600 nm (OD_600_) of 0.1 with enriched 7H9 medium. One hundred microliters of diluted cells per well were inoculated into clear flat-bottom 96-well plates containing enriched 7H9 medium with the assay compounds in a total volume of 100 µl per well. The assay plates were incubated for 1 week at 37°C under stationary conditions. The MIC for each strain was defined as the lowest concentration of a drug needed to inhibit bacterial growth via visual observation.

### Single-molecule analysis of MmpL5-GFP using TIRF microscopy.

Cells were grown in enriched 7H9 medium under shaking conditions at 37°C to the mid-logarithmic phase. The cultures were diluted by 50-fold using fresh enriched 7H9 medium and incubated at 37°C to the mid-logarithmic phase. Cells were harvested and washed twice with phosphate-buffered saline (PBS) containing 0.05% Tween 80 (PBS-T).

Cells were spotted onto a poly-l-lysine-coated coverslip chamber, washed three times with PBS-T, and observed via TIRF microscopy (Olympus IX71, Tokyo, Japan) with a ×100 oil-immersion objective lens (total magnification = ×1,000). The image analysis and tracking of single foci were performed using ImageJ ver. 1.52a software (NIH, MD, USA). These analyses were conducted as described previously (31). In brief, the images were filtered using the rolling ball algorithm to subtract the background intensity. We defined a 350 × 350 nm^2^ region of interest (ROI) centered on a focus in the first frame of the images with the threshold appropriately adjusted (52). MSD was calculated as described in the literature (53). *D* was also calculated from the values of the averaged MSD-Δ*t* plots.

### Photobleaching analysis.

The photobleaching analysis was performed as described in the literature (31). Images were recorded using an exposure time of 33 ms via TIRF microscopy. The fluorescence intensity per frame of an ROI centered on a fluorescent focus was monitored. The edge detection method of nonlinear filtering (window = 10) was used to identify photobleaching steps (54, 55).

### Immunoblotting.

The cells, which were cultured using same methods described for TIRF observation, were harvested and resuspended in 1 ml of sonication buffer (50 mM Tris-HCl, 200 mM NaCl, 10% glycerol). The precipitates were fractured via sonication (Bioruptor UCD-250; Cosmobio, Tokyo, Japan) at 250 W for 7 min. These fractions were used in an immunoblotting assay using a monoclonal anti-GFP antibody (Nacalai Tesque, Kyoto, Japan) following sodium dodecyl sulfate (SDS)-PAGE with a laboratory-produced 15% acrylamide gel. Immune complexes were detected using horseradish peroxidase (HRP)-labeled anti-mouse IgG (Cell Signaling Technology, MA., USA) and the Immobilon Western Chemiluminescent HRP Substrate system (Merck Millipore, MA., USA).

### TEM.

The cells, which were cultured using the same methods described for TIRF observation, were harvested and washed with PBS-T. During prefixation, cell samples were treated with 2% glutaraldehyde and 2.5% paraformaldehyde in 30 mM HEPES buffer (pH 7.4) for 2 days at 4°C and stained with 0.2% tannin for 20 min. During postfixation, cells were treated with 1% osmium tetroxide solution containing 0.8% potassium ferricyanide for 1 h. The cells were dehydrated using a graded ethanol series (50, 70, 80, 90, 95, and 100%) at room temperature, followed by gradual infiltration with Spurr’s resin (Polysciences) over 2 days (41). Ultrathin sections (70-nm thickness) were cut using an ultramicrotome (EM UC 7; Leica, Wetzlar, Germany), mounted on grids, and stained with 4% uranyl acetate and lead citrate. The observations were performed using a transmission electron microscope (HT7700; Hitachi High Technologies, Tokyo, Japan) at 80 kV.

### Cell wall permeability assay.

The EtBr permeability assay was performed by measuring fluorescence intensity as described in the literature (56). Cells were grown in enriched 7H9 medium under shaking conditions at 37°C to the mid-logarithmic phase. The cell precipitates were washed twice with PBS-T and incubated with 1 µg ml^−1^ EtBr containing 100 µg ml^−1^ verapamil for 60 min at 37°C under stationary conditions. After washing cells twice with PBS-T, the fluorescence intensity was measured using a multimode microplate reader (Nivo 3S; PerkinElmer, MA, USA). Cells were incubated in 96-well black Fluoro plates (Thermo Fisher Scientific, MA, USA) containing enriched 7H9 medium with the assay reagents in a volume of 200 µl well^−1^, and the measurement was performed at different time points for a unit of time for each measurement of 5 min (excitation, 530 nm; emission, 570 nm). All data were normalized to the time zero value of each well and the bacterial cell density.

10.1128/mSphere.00518-20.4 TABLE S2 Primers used in this study. Download Table S2, DOCX file, 0.03 MB. Copyright © 2021 Yamamoto et al. 2021 Yamamoto et al. This content is distributed under the terms of the Creative Commons Attribution 4.0 International license.

## References

[1] WHO. 2019 Global tuberculosis report 2019. World Health Organization (WHO), Geneva, Switzerland.

[2] Hoffmann C, Leis A, Niederweis M, Plitzko JM, Engelhardt H 2008 Disclosure of the mycobacterial outer membrane: cryo-electron tomography and vitreous sections reveal the lipid bilayer structure. Proc Natl Acad Sci U S A 105:3963–3967. doi:10.1073/pnas.0709530105. 18316738 PMC2268800

[3] Zuber B, Chami M, Houssin C, Dubochet J, Griffiths G, Daffe M 2008 Direct visualization of the outer membrane of mycobacteria and corynebacteria in their native state. J Bacteriol 190:5672–5680. doi:10.1128/JB.01919-07. 18567661 PMC2519390

[4] Paulsen IT, Brown MH, Skurray RA 1996 Proton-dependent multidrug efflux systems. Microbiol Rev 60:575–608. doi:10.1128/MR.60.4.575-608.1996. 8987357 PMC239457

[5] Li XZ, Nikaido H 2009 Efflux-mediated drug resistance in bacteria: an update. Drugs 69:1555–1623. doi:10.2165/11317030-000000000-00000. 19678712 PMC2847397

[6] Nikaido H, Zgurskaya HI 2001 AcrAB and related multidrug efflux pumps of *Escherichia coli*. J Mol Microbiol Biotechnol 3:215–218. 11321576

[7] Nishino K, Yamaguchi A 2001 Analysis of a complete library of putative drug transporter genes in *Escherichia coli*. J Bacteriol 183:5803–5812. doi:10.1128/JB.183.20.5803-5812.2001. 11566977 PMC99656

[8] Murakami S, Nakashima R, Yamashita E, Matsumoto T, Yamaguchi A 2006 Crystal structures of a multidrug transporter reveal a functionally rotating mechanism. Nature 443:173–179. doi:10.1038/nature05076. 16915237

[9] Zgurskaya HI, Nikaido H 1999 Bypassing the periplasm: reconstitution of the AcrAB multidrug efflux pump of *Escherichia coli*. Proc Natl Acad Sci U S A 96:7190–7195. doi:10.1073/pnas.96.13.7190. 10377390 PMC22048

[10] Okusu H, Ma D, Nikaido H 1996 AcrAB efflux pump plays a major role in the antibiotic resistance phenotype of *Escherichia coli* multiple-antibiotic-resistance (Mar) mutants. J Bacteriol 178:306–308. doi:10.1128/jb.178.1.306-308.1996. 8550435 PMC177656

[11] Tekaia F, Gordon SV, Garnier T, Brosch R, Barrell BG, Cole ST 1999 Analysis of the proteome of *Mycobacterium tuberculosis* in silico. Tuber Lung Dis 79:329–342. doi:10.1054/tuld.1999.0220. 10694977

[12] Domenech P, Reed MB, Barry CE III. 2005 Contribution of the *Mycobacterium tuberculosis* MmpL protein family to virulence and drug resistance. Infect Immun 73:3492–3501. doi:10.1128/IAI.73.6.3492-3501.2005. 15908378 PMC1111821

[13] Pacheco SA, Hsu FF, Powers KM, Purdy GE 2013 MmpL11 protein transports mycolic acid-containing lipids to the mycobacterial cell wall and contributes to biofilm formation in *Mycobacterium smegmatis*. J Biol Chem 288:24213–24222. doi:10.1074/jbc.M113.473371. 23836904 PMC3745366

[14] Chalut C 2016 MmpL transporter-mediated export of cell-wall associated lipids and siderophores in mycobacteria. Tuberculosis (Edinb) 100:32–45. doi:10.1016/j.tube.2016.06.004. 27553408

[15] Viljoen A, Dubois V, Girard-Misguich F, Blaise M, Herrmann JL, Kremer L 2017 The diverse family of MmpL transporters in mycobacteria: from regulation to antimicrobial developments. Mol Microbiol 104:889–904. doi:10.1111/mmi.13675. 28340510

[16] De Rossi E, Ainsa JA, Riccardi G 2006 Role of mycobacterial efflux transporters in drug resistance: an unresolved question. FEMS Microbiol Rev 30:36–52. doi:10.1111/j.1574-6976.2005.00002.x. 16438679

[17] Belardinelli JM, Larrouy-Maumus G, Jones V, Sorio de Carvalho LP, McNeil MR, Jackson M 2014 Biosynthesis and translocation of unsulfated acyltrehaloses in *Mycobacterium tuberculosis*. J Biol Chem 289:27952–27965. doi:10.1074/jbc.M114.581199. 25124040 PMC4183827

[18] Andries K, Villellas C, Coeck N, Thys K, Gevers T, Vranckx L, Lounis N, de Jong BC, Koul A 2014 Acquired resistance of *Mycobacterium tuberculosis* to bedaquiline. PLoS One 9:e102135. doi:10.1371/journal.pone.0102135. 25010492 PMC4092087

[19] Wells RM, Jones CM, Xi Z, Speer A, Danilchanka O, Doornbos KS, Sun P, Wu F, Tian C, Niederweis M 2013 Discovery of a siderophore export system essential for virulence of *Mycobacterium tuberculosis*. PLoS Pathog 9:e1003120. doi:10.1371/journal.ppat.1003120. 23431276 PMC3561183

[20] Radhakrishnan A, Kumar N, Wright CC, Chou TH, Tringides ML, Bolla JR, Lei HT, Rajashankar KR, Su CC, Purdy GE, Yu EW 2014 Crystal structure of the transcriptional regulator Rv0678 of *Mycobacterium tuberculosis*. J Biol Chem 289:16526–16540. doi:10.1074/jbc.M113.538959. 24737322 PMC4047419

[21] Ioerger TR, O’Malley T, Liao R, Guinn KM, Hickey MJ, Mohaideen N, Murphy KC, Boshoff HI, Mizrahi V, Rubin EJ, Sassetti CM, Barry CE III, Sherman DR, Parish T, Sacchettini JC 2013 Identification of new drug targets and resistance mechanisms in *Mycobacterium tuberculosis*. PLoS One 8:e75245. doi:10.1371/journal.pone.0075245. 24086479 PMC3781026

[22] Milano A, Pasca MR, Provvedi R, Lucarelli AP, Manina G, Ribeiro AL, Manganelli R, Riccardi G 2009 Azole resistance in *Mycobacterium tuberculosis* is mediated by the MmpS5-MmpL5 efflux system. Tuberculosis (Edinb) 89:84–90. doi:10.1016/j.tube.2008.08.003. 18851927

[23] Zhang B, Li J, Yang X, Wu L, Zhang J, Yang Y, Zhao Y, Zhang L, Yang X, Yang X, Cheng X, Liu Z, Jiang B, Jiang H, Guddat LW, Yang H, Rao Z 2019 Crystal structures of membrane transporter MmpL3, an anti-TB drug target. Cell 176:636–648.e13. doi:10.1016/j.cell.2019.01.003. 30682372

[24] Du D, Wang Z, James NR, Voss JE, Klimont E, Ohene-Agyei T, Venter H, Chiu W, Luisi BF 2014 Structure of the AcrAB-TolC multidrug efflux pump. Nature 509:512–515. doi:10.1038/nature13205. 24747401 PMC4361902

[25] Deshayes C, Bach H, Euphrasie D, Attarian R, Coureuil M, Sougakoff W, Laval F, Av-Gay Y, Daffe M, Etienne G, Reyrat JM 2010 MmpS4 promotes glycopeptidolipids biosynthesis and export in *Mycobacterium smegmatis*. Mol Microbiol 78:989–1003. doi:10.1111/j.1365-2958.2010.07385.x. 21062372

[26] Briffotaux J, Huang W, Wang X, Gicquel B 2017 MmpS5/MmpL5 as an efflux pump in Mycobacterium species. Tuberculosis (Edinb) 107:13–19. doi:10.1016/j.tube.2017.08.001. 29050760

[27] Sandhu P, Akhter Y 2018 Evolution of structural fitness and multifunctional aspects of mycobacterial RND family transporters. Arch Microbiol 200:19–31. doi:10.1007/s00203-017-1434-6. 28951954

[28] Hartkoorn RC, Uplekar S, Cole ST 2014 Cross-resistance between clofazimine and bedaquiline through upregulation of MmpL5 in *Mycobacterium tuberculosis*. Antimicrob Agents Chemother 58:2979–2981. doi:10.1128/AAC.00037-14. 24590481 PMC3993252

[29] Villellas C, Coeck N, Meehan CJ, Lounis N, de Jong B, Rigouts L, Andries K 2017 Unexpected high prevalence of resistance-associated Rv0678 variants in MDR-TB patients without documented prior use of clofazimine or bedaquiline. J Antimicrob Chemother 72:684–690. 28031270 10.1093/jac/dkw502 PMC5400087

[30] Mikolosko J, Bobyk K, Zgurskaya HI, Ghosh P 2006 Conformational flexibility in the multidrug efflux system protein AcrA. Structure 14:577–587. doi:10.1016/j.str.2005.11.015. 16531241 PMC1997295

[31] Yamamoto K, Tamai R, Yamazaki M, Inaba T, Sowa Y, Kawagishi I 2016 Substrate-dependent dynamics of the multidrug efflux transporter AcrB of *Escherichia coli*. Sci Rep 6:21909. doi:10.1038/srep21909. 26916090 PMC4768149

[32] Kumar M, Mommer MS, Sourjik V 2010 Mobility of cytoplasmic, membrane, and DNA-binding proteins in *Escherichia coli*. Biophys J 98:552–559. doi:10.1016/j.bpj.2009.11.002. 20159151 PMC2820653

[33] Cowan AE, Koppel DE, Setlow B, Setlow P 2003 A soluble protein is immobile in dormant spores of *Bacillus subtilis* but is mobile in germinated spores: implications for spore dormancy. Proc Natl Acad Sci U S A 100:4209–4214. doi:10.1073/pnas.0636762100. 12646705 PMC404470

[34] Cowan AE, Olivastro EM, Koppel DE, Loshon CA, Setlow B, Setlow P 2004 Lipids in the inner membrane of dormant spores of Bacillus species are largely immobile. Proc Natl Acad Sci U S A 101:7733–7738. doi:10.1073/pnas.0306859101. 15126669 PMC419675

[35] Mika JT, Schavemaker PE, Krasnikov V, Poolman B 2014 Impact of osmotic stress on protein diffusion in *Lactococcus lactis*. Mol Microbiol 94:857–870. doi:10.1111/mmi.12800. 25244659

[36] Silhavy TJ, Kahne D, Walker S 2010 The bacterial cell envelope. Cold Spring Harb Perspect Biol 2:a000414. doi:10.1101/cshperspect.a000414. 20452953 PMC2857177

[37] Richard M, Gutierrez AV, Viljoen AJ, Ghigo E, Blaise M, Kremer L 2018 Mechanistic and structural insights into the unique TetR-dependent regulation of a drug efflux pump in *Mycobacterium abscessus*. Front Microbiol 9:649. doi:10.3389/fmicb.2018.00649. 29675007 PMC5895659

[38] Richard M, Gutierrez AV, Viljoen A, Rodriguez-Rincon D, Roquet-Baneres F, Blaise M, Everall I, Parkhill J, Floto RA, Kremer L 2018 Mutations in the MAB_2299c TetR regulator confer cross-resistance to clofazimine and bedaquiline in *Mycobacterium abscessus*. Antimicrob Agents Chemother 63:e01316-18. doi:10.1128/AAC.01316-18. 30323043 PMC6325171

[39] Varela C, Rittmann D, Singh A, Krumbach K, Bhatt K, Eggeling L, Besra GS, Bhatt A 2012 MmpL genes are associated with mycolic acid metabolism in mycobacteria and corynebacteria. Chem Biol 19:498–506. doi:10.1016/j.chembiol.2012.03.006. 22520756 PMC3370651

[40] Wright CC, Hsu FF, Arnett E, Dunaj JL, Davidson PM, Pacheco SA, Harriff MJ, Lewinsohn DM, Schlesinger LS, Purdy GE 2017 The *Mycobacterium tuberculosis* MmpL11 cell wall lipid transporter is important for biofilm formation, intracellular growth, and nonreplicating persistence. Infect Immun 85:e00131-17. doi:10.1128/IAI.00131-17. 28507063 PMC5520431

[41] Campodonico VL, Rifat D, Chuang YM, Ioerger TR, Karakousis PC 2018 Altered *Mycobacterium tuberculosis* cell wall metabolism and physiology associated with RpoB mutation H526D. Front Microbiol 9:494. doi:10.3389/fmicb.2018.00494. 29616007 PMC5867343

[42] Farrow MF, Rubin EJ 2008 Function of a mycobacterial major facilitator superfamily pump requires a membrane-associated lipoprotein. J Bacteriol 190:1783–1791. doi:10.1128/JB.01046-07. 18156250 PMC2258667

[43] Adams KN, Szumowski JD, Ramakrishnan L 2014 Verapamil, and its metabolite norverapamil, inhibit macrophage-induced, bacterial efflux pump-mediated tolerance to multiple anti-tubercular drugs. J Infect Dis 210:456–466. doi:10.1093/infdis/jiu095. 24532601 PMC4110457

[44] Amaral L, Spengler G, Martins A, Armada A, Handzlik J, Kiec-Kononowicz K, Molnar J 2012 Inhibitors of bacterial efflux pumps that also inhibit efflux pumps of cancer cells. Anticancer Res 32:2947–2957. 22753759

[45] Stover CK, de la Cruz VF, Fuerst TR, Burlein JE, Benson LA, Bennett LT, Bansal GP, Young JF, Lee MH, Hatfull GF 1991 New use of BCG for recombinant vaccines. Nature 351:456–460. doi:10.1038/351456a0. 1904554

[46] Nakata N, Kai M, Makino M 2012 Mutation analysis of mycobacterial *rpoB* genes and rifampin resistance using recombinant *Mycobacterium smegmatis*. Antimicrob Agents Chemother 56:2008–2013. doi:10.1128/AAC.05831-11. 22252831 PMC3318355

[47] Nakata N, Kai M, Makino M 2011 Mutation analysis of the *Mycobacterium leprae folP1* gene and dapsone resistance. Antimicrob Agents Chemother 55:762–766. doi:10.1128/AAC.01212-10. 21115799 PMC3028759

[48] Bardarov S, Bardarov S, Pavelka MS, Sambandamurthy V, Larsen M, Tufariello J, Chan J, Hatfull G, Jacobs WR 2002 Specialized transduction: an efficient method for generating marked and unmarked targeted gene disruptions in *Mycobacterium tuberculosis*, *M. bovis* BCG and *M. smegmatis*. Microbiology 148:3007–3017. doi:10.1099/00221287-148-10-3007. 12368434

[49] van Kessel JC, Hatfull GF 2007 Recombineering in *Mycobacterium tuberculosis*. Nat Methods 4:147–152. doi:10.1038/nmeth996. 17179933

[50] Shetty A, Dick T 2018 Mycobacterial cell wall synthesis inhibitors cause lethal ATP burst. Front Microbiol 9:1898. doi:10.3389/fmicb.2018.01898. 30158918 PMC6104191

[51] Wiegand I, Hilpert K, Hancock RE 2008 Agar and broth dilution methods to determine the minimal inhibitory concentration (MIC) of antimicrobial substances. Nat Protoc 3:163–175. doi:10.1038/nprot.2007.521. 18274517

[52] Leake MC, Chandler JH, Wadhams GH, Bai F, Berry RM, Armitage JP 2006 Stoichiometry and turnover in single, functioning membrane protein complexes. Nature 443:355–358. doi:10.1038/nature05135. 16971952

[53] Kusumi A, Sako Y, Yamamoto M 1993 Confined lateral diffusion of membrane receptors as studied by single particle tracking (nanovid microscopy). Effects of calcium-induced differentiation in cultured epithelial cells. Biophys J 65:2021–2040. doi:10.1016/S0006-3495(93)81253-0. 8298032 PMC1225938

[54] Leake MC, Wilson D, Gautel M, Simmons RM 2004 The elasticity of single titin molecules using a two-bead optical tweezers assay. Biophys J 87:1112–1135. doi:10.1529/biophysj.103.033571. 15298915 PMC1304451

[55] Chung SH, Kennedy RA 1991 Forward-backward non-linear filtering technique for extracting small biological signals from noise. J Neurosci Methods 40:71–86. doi:10.1016/0165-0270(91)90118-j. 1795554

[56] Chuang YM, Bandyopadhyay N, Rifat D, Rubin H, Bader JS, Karakousis PC 2015 Deficiency of the novel exopolyphosphatase Rv1026/PPX2 leads to metabolic downshift and altered cell wall permeability in *Mycobacterium tuberculosis*. mBio 6:e02428-14. doi:10.1128/mBio.02428-14. 25784702 PMC4453511

