# Supplementary figures and images for: Coexpression of MmpS5 and MmpL5 Contributes to Both Efflux Transporter MmpL5 Trimerization and Drug Resistance in Mycobacterium tuberculosis
Source: mSphere. 2021 Jan 6;6(1):e00518-20. doi: 10.1128/mSphere.00518-20 (PMC7845600; doi:10.1128/mSphere.00518-20)

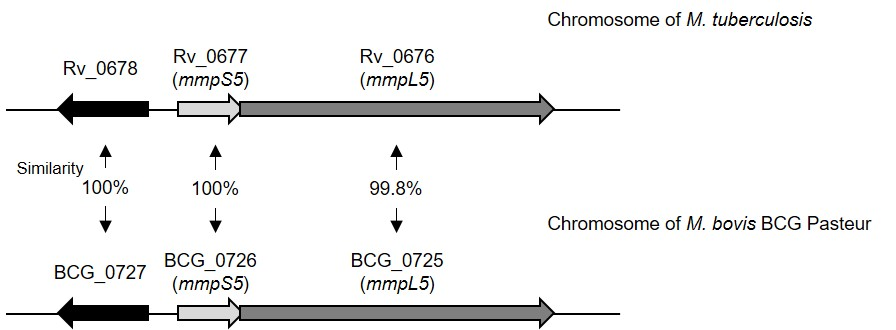

Supplement: FIG S1 [file mSphere.00518-20-sf001.tif]

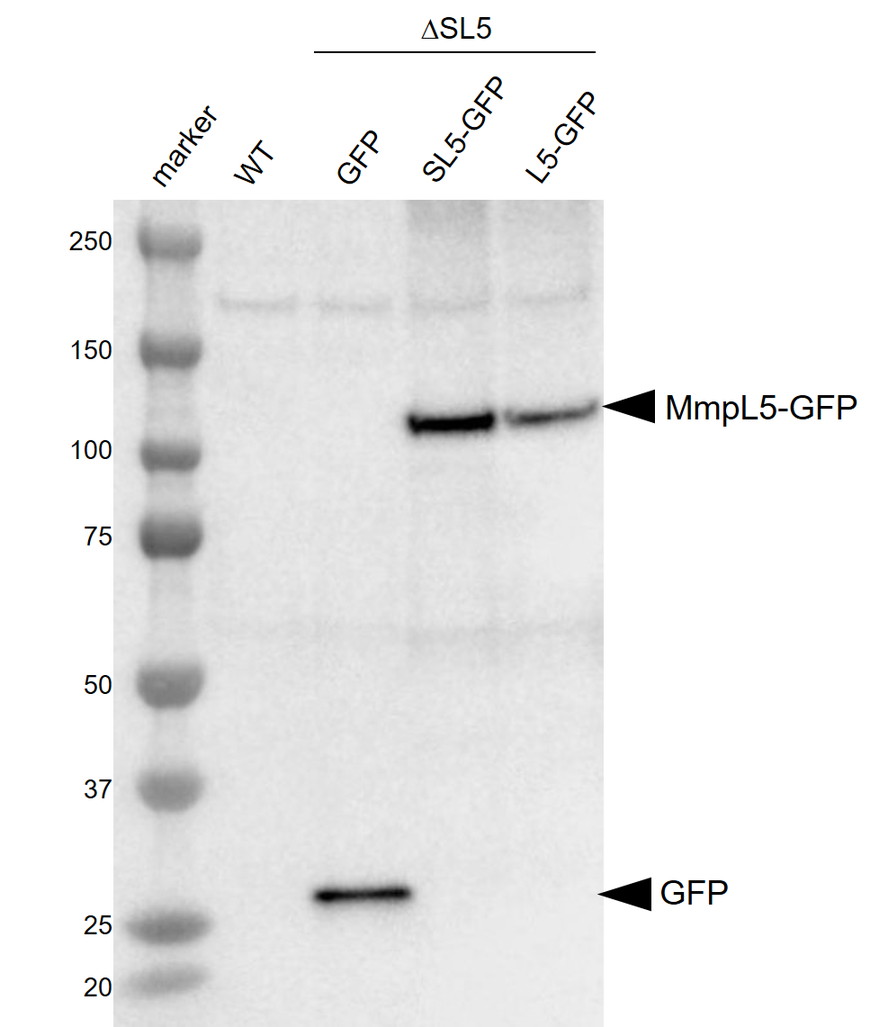

Supplement: FIG S2 [file mSphere.00518-20-sf002.jpg]
