# Supplementary material for: Coexpression of MmpS5 and MmpL5 Contributes to Both Efflux Transporter MmpL5 Trimerization and Drug Resistance in Mycobacterium tuberculosis
Source: mSphere. 2021 Jan 6;6(1):e00518-20. doi: 10.1128/mSphere.00518-20 (PMC7845600; doi:10.1128/mSphere.00518-20)
Supplement: TABLE S1 [file mSphere.00518-20-st001.docx]

|  | Relevant characteristics | Parent | Source or reference |
| --- | --- | --- | --- |
| **Strains** |  |  |  |
| *M. bovis* BCG Pasteur strain | wild-type |  |  |
| NNB001 | ∆*mmpS5-L5-*BCG_0727::*Hyg* | WT BCG Pasteur strain | This study |
| YKN80 | ∆*mmpS5-L5*::*Hyg* | WT BCG Pasteur strain | This study |
|  |  |  |  |
| **Plasmids** |  |  |  |
| pNN301 | *Escherichia coli*-*Mycobacterium* shuttle vector | pMV361 | Nakata *et al.*, 2012 |
| pDS1050 | Gly3-linked-EGFP fusion vector plasmid (C-terminal) | pTrcHisB | Hiremath *et al.*, 2015 |
| pKRB1 | Gly3-linked-EGFP fusion vector plasmid (C-terminal) | pNN301 | This study |
| pKRB29 | MmpL5-EGFP | pNN301 | This study |
| pKRB30 | MmpL5 | pNN301 | This study |
| pKRB32 | MmpS5-L5-EGFP | pNN301 | This study |
| pKRB34 | MmpS5-L5 | pNN301 | This study |
| pJV53 | Che9c genes 60-61 | pLAM12 | van Kessel & Hatfull, 2007 |
| pYUB854 | hygromycin-resistance gene | – | Bardarov *et al.*, 2002 |
|  |  |  |  |
