# Supplementary material for: Coexpression of MmpS5 and MmpL5 Contributes to Both Efflux Transporter MmpL5 Trimerization and Drug Resistance in Mycobacterium tuberculosis
Source: mSphere. 2021 Jan 6;6(1):e00518-20. doi: 10.1128/mSphere.00518-20 (PMC7845600; doi:10.1128/mSphere.00518-20)
Supplement: TABLE S2 [file mSphere.00518-20-st002.docx]

| Primer name | Sequence | Note |
| --- | --- | --- |
| G3-egfp-F(Sal I+ac) | acgcgtcgacacggcggaggcAGCAAGGGCGAGGAGCTGTTC | pKRB1 cloning primer |
| G3-egfp-R(Hpa I) | acgcgttaacTTACTTGTACAGCTCGTCCATGCCGAGAG | pKRB1 cloning primer |
| mmpS5-SD-NdeI-F | attgcggatccagctgcagaattcTGAggaggaatctcCATATGattggaactctcaagcgtgcctggataccg | pKRB32 and pKRB34 cloning primer |
| mmpL5-R(ClaI+a)SLiCE | tccgccgtgtcgacatcgataGACCAAGGCGAAGGTCCGTGCCGATGCCGG | pKRB29, 30, 32, 34 cloning primer |
| mmpL5-SD-NdeI-F | attgcggatccagctgcagaattcTGAggaggaatctcCATATGatcgtgcaaaggacagctgcgccgacgggc | pKRB29 and pKRB30 cloning primer |
| upF | GCCCTTAAGGCTCACCGATCCGATGTTGC | construction of NNB001 primer |
| upR | GCCTCTAGATCTGAGCCGTACCCGCCTAA | construction of NNB001 primer |
| udnF | GCCAAGCTTGCAACCTCGCAATCTGACCG | construction of NNB001 primer |
| dnR | GCCACTAGTATGCACGTACGCTCAATCAA | construction of NNB001 primer |
| mmpS5 up1000-F | CCCGCGACCGCCACCACCACGACAGCGACGAGC | construction of YKN80 primer |
| mmpS5 up33+AflII30-R | gctctggtaccctctagtcaaggccttaagCGCCCTCCGCCTCTGCCGCATGAAGTTCACGCC | construction of YKN80 primer |
| pYU854 Hyg-AflII30-F | cttaaggccttgactagagggtaccagagc | construction of YKN80 primer |
| pYU854 Hyg-SpeI30-R | actagtgggcagatcttcgaatgcatcgcg | construction of YKN80 primer |
| mmpL5 down33+SpeI-F | cgcgatgcattcgaagatctgcccactagtGCCGTACCCGCCTAAAGCGTGTCGCCCTGTTCG | construction of YKN80 primer |
| mmpL5 down1000-R | GACATGGCAGCGGCCACCGACATGCCCGGGCG | construction of YKN80 primer |
